# Joint Tissue Protective and Immune-Modulating miRNA Landscape of Mesenchymal Stromal Cell-Derived Extracellular Vesicles under Different Osteoarthritis-Mimicking Conditions

**DOI:** 10.3390/pharmaceutics14071400

**Published:** 2022-07-02

**Authors:** Enrico Ragni, Carlotta Perucca Orfei, Federico Sinigaglia, Laura de Girolamo

**Affiliations:** Laboratorio di Biotecnologie Applicate all′Ortopedia, IRCCS Istituto Ortopedico Galeazzi, Via R. Galeazzi 4, 20161 Milano, Italy; enrico.ragni@grupposandonato.it (E.R.); carlotta.perucca@grupposandonato.it (C.P.O.); federico.sinigaglia@grupposandonato.it (F.S.)

**Keywords:** mesenchymal stromal cells, extracellular vesicles, miRNAs, osteoarthritis, cartilage, synovium, immune cells

## Abstract

In regenerative medicine related to orthopedic conditions, mesenchymal stromal cells (MSCs) and their extracellular vesicles (EVs) have been proposed as innovative clinical options. The definition of EV-shuttled signals and their modulation under orthopedic settings, such as osteoarthritis (OA), is crucial for MSC-related research, both for basic science and for use in clinical settings, either as therapeutics or as producers of cell-free products such as EVs or secretome. The objective of this work is to compare the literature available on high-throughput EV-miRNA data obtained from adipose-derived MSCs (ASCs) in standard conditions or cultured in high levels of IFNγ, low-level inflammatory conditions mimicking OA synovial fluid (SF), and OA-SF. The first result was that both IFNγ and low-level inflammatory treatment led to an increase, whereas SF led to a reduction in EV release. Second, more than 200 EV-miRNAs were found to be shared across the different conditions. After a bioinformatics search through experimentally validated and OA-related targets, pathways and tissues, several miRNAs resulted in the restoration of cartilage and synovium stability and the homeostasis of inflammatory cells, including macrophages, promoting their switch towards an M2 anti-inflammatory phenotype. Third, IFNγ and especially SF culturing were able to modulate the overall EV-miRNA fingerprint, although the main molecular messages related to OA resulted conserved between treatments with the majority of modulations within 2-fold range. In conclusion, ASC EV-miRNAs may be modulated in their overall landscape by OA-related culturing conditions albeit resulted largely stable in their specific OA-protective signals allowing for a faster clinical translation of these new cell-free therapies for joint diseases.

## 1. Introduction

Osteoarthritis (OA) is a degenerative joint disease that triggers the gradual degradation of articular cartilage until its complete loss, alongside osteophyte formation, subchondral bone changes, and meniscal alterations [1]. Often, OA patients experience synovial inflammation [2] with the involvement of resident inflammatory cells, such as synovial macrophages [3]. None of the available therapies are able to modify disease progression or prevent final joint replacement in the advanced disease stage [4]. Therefore, there is an urgent need for disease-modifying therapies able to promote both cartilage restoration and inflammation reduction in order to elude or at least delay the need for joint replacement.

In this context, mesenchymal stromal cells (MSCs) have emerged as an attractive option due to their regenerative and immunomodulatory features [5] mainly ascribed to their released factors [6], both free and conveyed within extracellular vesicles (EVs). Consistently, the recent clinical trials which used MSC-based treatments in patients with OA were safe, showed an improvement in clinical outcomes [7], and demonstrated improvement [8] or no progression [9] in cartilage loss. Intriguingly, when cartilage morphology and collagen content did not change relative to baseline, cartilage catabolic biomarkers and synovitis were significantly lower with inflammatory macrophages and cytokine levels decreased in the synovial fluid after MSC injection [10]. Of note, the authors reported that in vitro IFNγ-treated MSC secretory profiles correlated with clinical outcomes [10] using the same MSC batches, confirming the crucial role of released factors for MSCs’ therapeutic properties. This is consistent with several in vitro data reporting how IFNγ and other inflammatory cytokines present in OA synovial fluid as IL1β and TNFα, enhance MSCs’ immunosuppressive properties, and promote the M2 polarization of macrophages through the release of soluble mediators [11].

Under this paradigm, MSC-EVs have been suggested as a cell-free approach for OA treatment. In several animal models, MSC-EVs were able to reduce inflammation and macrophage activity, as well as being able to restore cartilage homeostasis, volume and thickness [12]. Due to these preclinical results, in September 2021 a clinical trial started assessing the safety and efficacy of MSC-EVs in patients with knee OA (https://clinicaltrials.gov/ct2/show/NCT050601077, accessed on 1 May 2022). Therefore, the time is ripe to understand the molecular mechanisms beyond MSC-EV efficacy for OA treatment, allowing broad spectrum and effective translatability into clinical everyday practice. The loss and gain of function approaches in vivo have shown how cartilage protective and immunomodulatory MSC-EV features greatly rely on embedded miRNAs [13,14]. Consistently, our group has shown that adipose derived-MSC (ASC) EVs shuttle several miRNAs with cartilage protective and anti-inflammatory properties, and that these features are present when cells are cultured under standard conditions [15], high levels of IFNγ [15] (proposed to improve EVs’ potential), OA synovial fluid-like levels of IFNγ/TNFα/IL1β [16], and eventually OA synovial fluid [17]. These results raise questions about whether EV-miRNA fingerprints may diverge depending on the environmental surrounding, or whether standard cultures may reflect conditions similar to those ASCs encounter when administrated to patients.

To answer these questions, in this report we compared previously released ASC-EVs miRNA datasets to identify abundant and modulated players, with ASCs isolates used in the abovementioned studies identical, as well as the technical workflow and the analytical platforms. Results of this study will shed light on the differences between culturing conditions that will allow both identifying the setting producing ASC-EVs with improved OA-therapeutic potential and evaluating whether data obtained under standard culturing conditions may reliably reflect those from ASCs injected to patients.

## 2. Materials and Methods

### 2.1. Ethics Statement

The study was performed at IRCCS Istituto Ortopedico Galeazzi. Institutional Review Board approval (San Raffaele Hospital Ethics Committee approval on 16 December 2020, registered under number 214/int/2020) was granted before the beginning of the study.

### 2.2. Data Retrieval

Raw data were retrieved from the repository and supplementary material of previously published reports [15,16,17] that used the same ASC isolates from three female donors (54 ± 8 years old) undergoing liposuction. For publications [15,16], raw data related to donors 1, 2 and 3 were selected, since out of the 4 discussed donors in these two publications only the first 3 were used in publication [17]. Therefore, we selected the same donors that appeared in the 3 different publications to avoid inter-donor variability between studies. Additionally, to reduce batch effects, ASCs used for the different experiments described in the source manuscripts were generated from an original cell culture that was frozen in several aliquots, each used for a different treatment. The previously published raw data were newly analyzed in this manuscript. Viability, dimensional and release data for cells and EVs were implemented with the other two donors (both female, 60 and 61 years old). As an overview of the biological procedures described in the source manuscripts, in control medium of DMEM supplemented with 10% FBS (hereafter named C), stimuli were IFNγ (10 ng/mL) (I), TNFα (5 pg/mL) + IL1β (10 pg/mL) + IFNγ (40 pg/mL) (OA) and 50% OA synovial fluid (SF). To reduce another type of batch effects given by different experiments, the same medium with FBS and supplements was used. ASCs’ size and viability were detected with a Tali Image-based Cytometer (Thermo Fisher Scientific, Waltham, MA, USA) while morphology images were acquired with an IX71 microscope (Olympus CO Ltd., Tokyo, Japan). ASC flow cytometry was performed with a Cytoflex flow cytometer (Beckman Coulter, Fullerton, CA, USA) after staining with anti CD90-FITC (REA897), CD73-PE (REA804) and CD45-PEVIO770 (REA747) antibodies (Miltenyi, Bergisch Gladbach, Germany) following the manufacturer’s instructions. EVs were analyzed in FBS-free culture medium after 48 h of release in the same experimental conditions of approximately 90% confluence and 0.068 mL per cm^2^ culture flask. EVs were detected by both the NanoSight LM10-HS system (NanoSight Ltd., Amesbury, UK) and a CytoFLEX flow cytometer (Beckman Coulter, Brea, CA, USA) after carboxyfluorescein succinimidyl ester (CFSE) labelling and further staining with anti-CD9/63/81 (312107, 353007, 349509, BioLegend, San Diego, CA, USA).

As previously mentioned, EV-miRNA raw data were also retrieved from previous publications, repository or supplementary materials [15,16,17]. EV-miRNA raw amplification values were obtained as specified in the Materials and Methods sections of the source publications, using the same pipeline and technology. Briefly, to avoid batch effects in sample handling and preparation, the same protocol was followed with the same instrumentation and, after conditioned medium centrifugation at 4 °C for 15 min at 1000× *g* and 2000× *g* and twice at 4000× *g* to remove broken cells and debris, EVs were obtained with ultracentrifugation (100,000× *g*, 9 h, 4 °C) and trizol was used to dissolve the EV pellets, followed by RNA isolation with miRNeasy and RNeasy CleanUp Kits (Qiagen, Hilden, Germany). Before each RNA extraction, a non-human synthetic miRNA spike-in (6 pg, Arabidopsis thaliana ath-miR-159a) was added to monitor the technical variability during the whole detection procedure and to equalize the A and B panels of the OpenArray^®^ platform (Thermo Fisher Scientific). This also allowed us to reduce the batch effects for the data generated in the different experiments analyzed using ath-miR-159a as an internal technical control for all analyzed samples. Standard reverse transcription was used to obtain cDNAs, with preamplification performed with A and B independent kits, followed by real-time RT-PCR analysis with the QuantStudio™ 12 K Flex OpenArray^®^ Platform (QS12KFlex). miRNA expression data from A and B miRNA panels, covering 754 human miRNA sequences from the Sanger miRBase v21, were analyzed with the Expression Suite Software (Thermo Fisher Scientific). Each panel was designed to provide specificity for only the mature miRNA targets. A miRNA call was considered as positive only when amplification values were present in all three donors and negative when amplification values were absent in all three donors for a given condition. When single calls were randomly missing, the miRNA was not considered for further analysis. In the Thermo system, the amplification value is described as C_RT_, which stands for “cycle relative threshold”. The C_RT_ method accounts for the low reaction volumes and associated differences in fluorescence levels by analyzing the amplification curve from each through-hole individually. This method has proven to be more robust for analyzing data that are generated with the OpenArray plates containing 3072 through-holes that enable very low-volume (33 nL) reactions. Unlike the classical C_T_ method, which considers all the curves for a specific target to determine the threshold, the C_RT_ method sets a threshold for each curve individually that is based on the shape of the amplification curve, regardless of the height or variability of the curve in its early baseline fluorescence. The method allows lower variation across replicate samples while maintaining the same dynamic range.

### 2.3. miRNA Data Normalization

Normalization was newly performed using the global normalization procedure [18] with some modifications. As a general principle, the global mean normalization method is valid for miRNA profiling studies in which a large number and unbiased set of genes are measured, as in the present study. This method is based on the assumption that only a minority of miRNAs are differentially expressed in samples obtained from related cells or tissues, again as in the herein presented study. Briefly, after the identification of miRNAs to be analyzed across samples, for each miRNA the median C_RT_ value among all donors and conditions was obtained, followed by the identification of miRNAs lying in the first quartile of expression. This boundary was arbitrarily chosen since low-expressed miRNAs may generate high C_RT_ values with stochastic variation. In this group of abundant molecules, miRNAs with standard deviation (SD) > 1 in at least one condition were removed. Eventually, the median C_RT_ value obtained from the remaining miRNAs for each donor was used on the entire dataset as a classical reference gene for normalization, using the donor with the lowest median as the milestone.

### 2.4. miRNA Target Identification and Biological Process Identification

miRNAs under analysis were analyzed with miRTarBase v 8.0 (https://mirtarbase.cuhk.edu.cn/~miRTarBase/miRTarBase_2022/php/index.php, accessed on 21 February 2022) [19]. Only miRNA–mRNA interactions supported by strong experimental evidence were considered. Retrieved targets were analyzed with the Panther v 16.0 functional classification system (http://www.pantherdb.org/, accessed on 22 February 2022) [20] to identify biological processes (BPs).

### 2.5. Assessment of Reference Gene (RG) Stability

miRNA expression stability was evaluated according to four gold-standard statistical approaches: BestKeeper [21], geNorm [22], NormFinder [23], and the comparative delta-Ct method [24]. geNorm, NormFinder, and the delta-Ct method use transformed Ct values of (1 + E) − ΔCt, while BestKeeper uses Ct values directly. The ranking of the RGs according to their stability was generated by each algorithm and the overall performance of the miRNA RGs was evaluated by combining the results of the four approaches through a global ranking obtained as the geometric mean of the rankings given by each analysis [25].

### 2.6. Statistical Analyses

Statistical analyses were performed using GraphPad Prism Software version 5 (GraphPad, San Diego, CA, USA). The comparison between datasets using the control as the milestone was performed using ANOVA with Holm multiple comparison with the significance level set at *p*-value ≤ 0.05. When comparing miRNA datasets, adjusted *p*-value was calculated with Bonferroni correction considering the number of comparisons to limit the family error rate. The Pearson correlation coefficient (R^2^) was estimated to determine the linear association between the conditions. The outcome results were interpreted according to the degree of association [26].

Principal component analysis (PCA) and hierarchical clustering were conducted using normalized C_RT_ values with the ClustVis package (https://biit.cs.ut.ee/clustvis/, accessed on 18 February 2022) [27]. After row centering, maps were generated using the following settings for both the row and column clustering distances and methods: correlation and average, respectively.

## 3. Results

### 3.1. ASC and EV Characteristics

ASC size (mean ± SD) was found to be almost identical in control (C, 9.8 µm ± 0.8 µm) and low cytokine (OA, 9.6 µm ± 1.0 µm) conditions, while size was smaller under high IFNγ (I, 8.6 µm ± 0.5 µm) and synovial fluid (SF, 9.0 µm ± 0.0 µm) treatments. Morphology was identical and fibroblast-like for C, OA and I conditions, while it was more squared after SF treatment (Supplementary Figure S1). Viability was 93.0% ± 0.6% (C), 94.3% ± 1.3% (OA), 94.0% ± 1.0% (I) and 94.0% ± 1.6% (SF). In all conditions, ASCs were positive for CD73 (all conditions 100% ± 0%) and CD90 (96% ± 1% for C, 98% ± 1% for OA, 96% ± 1% for I and 100% ± 0% for SF) MSC markers, and negative for the hematological marker CD45 (all conditions 0% ± 0%).

**Figure 1:**
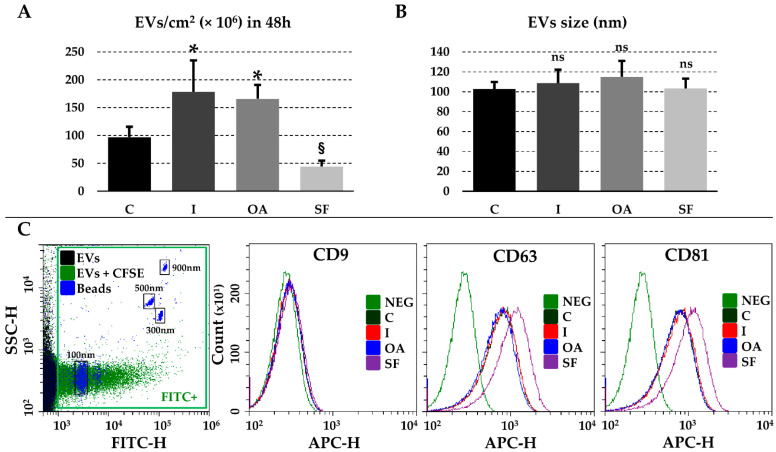
ASC-EV characterization. (**A**) Number of EVs secreted per cm^2^ in 48 h. § stands for *p*-value ≤ 0.1, * for *p*-value ≤ 0.05, *n* = 5 for C, I and OA samples, and *n* = 3 for SF samples, significance calculated vs. C using ANOVA test with Holm multiple comparison. (**B**) Mean particle size analysis from NTA data. ns stands for not significant, *n* = 3, significance calculated vs. C using Student *t*-test with significance level set at *p*-value ≤ 0.05. (**C**) Immunophenotype of released CFSE-labeled EVs after their gating as positive events in the FITC-H channel. FITC-fluorescent nanometric beads of predetermined size (100, 300, 500 and 900 nm) are shown in blue. CD9/63/81 staining is detected in the APC-H channel due to APC-conjugation of the respective antibodies. NEG refers to unstained CFSE-EVs and only the NEG plot for C condition is shown for clarity. One representative donor is shown.

The number of released particles per cm^2^ of cell culture surface in 48 h was (× 10^6^, mean ± SD) 97.2 ± 18.5 (C), 178.3 ± 56.4 (I), 165.9 ± 24.7 (OA) and 44.4 ± 10.3 (SF) (Figure 1A). Therefore, in vitro inflammatory conditions led to a significant (*p*-value ≤ 0.05) increase (ratio of 1.8 for I and 1.7 for OA with respect to C) while synovial fluid led to a consistent (*p*-value of 0.0826) reduction (ratio of 0.5 with respect to C) in EV secretion. Dimensional analysis did not show significant differences in terms of size (Figure 1B) with modes (nm, mean ± SD, *n* = 3) of 103 ± 7 (C), 109 ± 13 (I), 115 ± 16 (OA) and 103 ± 10 (SF). Regarding EV markers, all particles were strongly positive for CD63 and CD81 (Figure 1C), with similar values for C, I and OA (~80% positive events) and a 10% increase under SF (*p*-value of 0.0261 and 0.0466 for CD63 and CD81, respectively). The complete peak shift suggests the presence of CD63 and CD81 in the whole populations. Eventually, CD9 was very weak (from 4% to 6%) in all conditions (Figure 1C).

### 3.2. EV-Associated miRNAs

**Figure 2:**
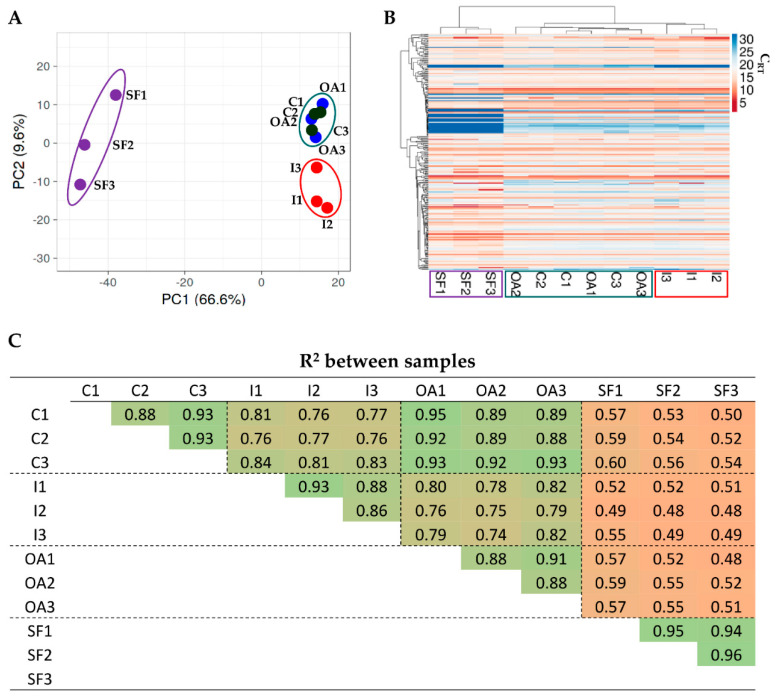
EV-miRNA fingerprint analysis between conditions. (**A**) Principal component analysis of normalized C_RT_ values of miRNAs. X and Y axis show principal component 1 and principal component 2 which explain 66.6% and 9.6% of the total variance. (**B**) Heat map of hierarchical clustering analysis of normalized C_RT_ values of detected miRNAs with sample clustering tree at the top. The color scale reflects the absolute expression levels: red shades = high expression levels and blue shades = low expression levels. Missed calls were set as C_RT_ = 40. (**C**) Correlation analysis of the C_RT_ values of miRNAs between samples after global mean normalization. A color scale relative to the amount of the R^2^ value is presented. Green boxes for higher R^2^ values, red boxes for lower R^2^ values.

In total, 252 miRNAs were detected in C and OA, 249 in I, and 223 in SF conditions (Supplementary Table S1). Two-hundred and twenty-three miRNAs were present in all conditions. As shown in Figure 2A, principal component analysis (PCA) was first performed for the expression profiles of EV-miRNAs that could largely distinguish SF samples and, to a much smaller extent, I samples from C and OA conditions that were coupled according to the donor (Figure 2A). This was confirmed by an unsupervised hierarchical clustering of the expression profiles for the differentially expressed miRNAs, revealing again a distinct expression signature of SF samples that lay under a distinct node (Figure 2B). Furthermore, I samples, although under the same original node, clustered separately from C and OA ones that were paired according to the donors. The clustering pattern was conserved when only the 223 shared miRNAs were considered reducing the influence of missing values in separating single conditions (data not shown). These results were confirmed by inter-condition correlation analysis (Figure 2C), where SF samples always gave very low R^2^ values (around 0.5) when compared to their counterparts in the other conditions. Again, C and OA samples gave similar results (R^2^ around 0.9), while I samples, although in a frame of similarity with C and OA, had lower values (R^2^ around 0.8). Notably, consistent intra-condition homogeneity between samples in each condition emerged, allowing us to average EV miRNA C_RT_ values for further analyses.

To attribute a biological significance to detected EV miRNAs, several parameters were considered. Initially, the first quartile of expression was identified after C_RT_ median calculation since, in MSC-EVs, even for abundant miRNAs no more than one copy per EV is present [28], and a minimal ratio of 100 EVs per target cell is needed to grant efficient miRNA transfer [29]. This led to a list of 63 miRNAs, covering 96.1% of the detected genetic message (Supplementary Table S2). Sifting experimentally validated miRNA–mRNA interactions for each of the first-quartile miRNAs (Supplementary Table S3), and 1364 univocal genes were identified (Supplementary Table S4). To reliably frame stable or differential expression, those miRNAs with a C_RT_ SD > 1 in at least one of the conditions were excluded. This led to 19 differentially expressed molecules (fold > 2 or <0.5, adjusted *p*-value ≤ 0.05) with respect to C conditions (Table 1): 3 downregulated miRNAs in the I samples targeting 172 genes (Supplementary Table S4); 13 downregulated (418 targets) and 3 upregulated miRNAs (97 targets) in the SF samples. To remove conflicting regulations, SF-specific down/upregulated miRNA targets were cleared of those present in both lists, resulting in 395 and 74 univocal targets for down and upregulated miRNAs, respectively (Supplementary Table S4).

**Figure 3:**
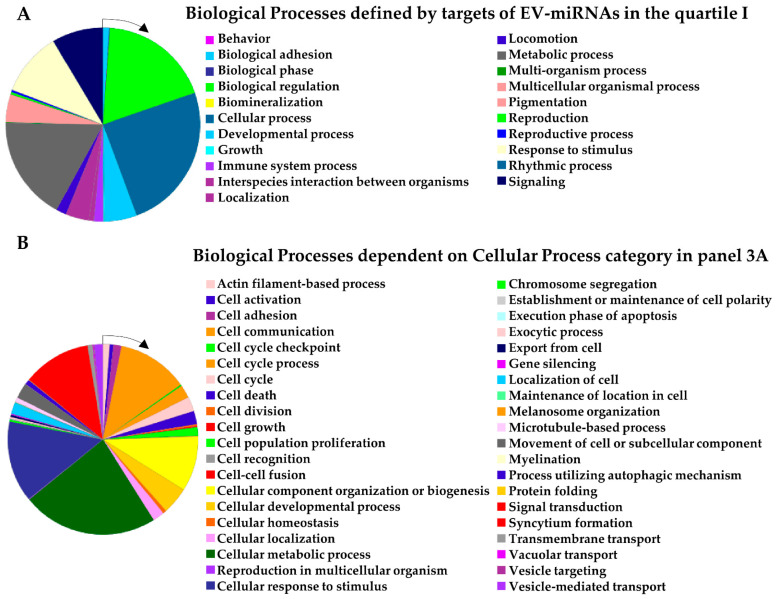
Biological processes (BPs) of first-quartile EV-miRNA univocal targets. (**A**) BPs defined by 1364 univocal targets of first-quartile EV-miRNAs obtained after median C_RT_ calculation of all conditions. (**B**) BPs defining the “cellular process” BP in panel A.

Eventually, to give an overview of the first-quartile miRNA targets, gene ontology analysis was performed. Sifting 1364 univocal targets, out of 21 categories, the top 3 biological processes (BPs) were found to be: cellular processes (948 genes, GO:0009987), biological regulation (715, GO:0065007) and metabolic processes (670, GO:0008152) (Figure 3A). Under the cellular process term, out of 40 BPs, the top 3 were cellular metabolic process (632, GO:0044237), cellular response to stimulus (378, GO:0051716) and cell communication (332, GO:0007154) (Figure 3B). Notably, an almost identical pattern of BPs for both number and terms was obtained with the target lists obtained for the differentially expressed miRNAs, although fewer genes were analyzed (Supplementary Figure S2). These results emphasize how the EV-miRNA regulation of disease-specific processes, possibly tuned by miRNA differential expression, can be identified only when a framed target dataset is sifted.

### 3.3. Target and Effect Prediction of EV-miRNAs on OA-Related Tissues

To dissect the effect of EV-miRNAs and their modulation in the OA setting, the first step was to compare the 1364 validated targets with cartilage and synovia-dependent molecular regulators of OA progression [30] (Table 2). This allowed for the identification of OA-related molecules targeted by first-quartile miRNAs and their total genetic weight, alongside the modulation of the main contributor. With respect to cytokines and chemokines involved in inflammation and extracellular matrix (ECM) homeostasis, EV-miRNAs target pro-inflammatory IL1α/β, IL6, IL18 and TNFα and ECM-erosive CCL5 and CXCL12. SF led to the downregulation of hsa-miR-125b-5p and hsa-miR-26a-5p, targeting TNFα and IL6, as well as the upregulation of hsa-miR-214-3p, targeting CCL5. IFNγ treatment strongly reduced hsa-miR-221-3p, the regulator of CXCL12. Notably, TNFα, IL1β and CXCL12, which are crucial for OA progression, were the most heavily targeted transcripts. With respect to growth factors, EV-miRNAs preferential targets were related to destructive molecules, such as TGFB1 and TGFB2, FGF1, CTGF and HGF. SF reduced hsa-miR-30c-5p, targeting CTGF. Moreover, VEGFA and ANGPT2, involved in abnormal angiogenesis during OA, were targeted by EV-miRNAs, with SF leading to a reduction in the ANGPT2 regulator hsa-miR-125b-5p. EV-miRNAs also targeted a few protective molecules, such as IGF1 and IGF2, the latter being the main target of SF-impaired hsa-miR-125b-5p. Eventually, the EV-miRNAs targeted several proteases and activators that were involved in ECM degradation, with MMP1/2/14, APC, PLAU/PLAT having the strongest regulation. After SF, the downregulation of hsa-miR-125b-5p may predict reduced inhibition for APC and MMP2, while the upregulation of hsa-miR-193b-3p may predict a stronger targeting of PLAU. Again, few ECM protective molecules were targeted, mainly TIMP3. Overall, for the three categories under consideration, destructive molecules were largely more targeted than protective ones.

The second step was to compare the first-quartile EV-miRNAs with those reported to be directly involved at different levels in OA progression and related to cartilage [31], synovia [32], and inflammatory macrophages [33] (Table 3) expressing many of the previously described cytokines/chemokines. Regarding cartilage, 15 protective and 7 destructive miRNAs were identified, tipping the balance towards cartilage recovery with the overall genetic weight of 47.38% vs. 10.17%. The most important contributors were (i) hsa-miR-24-3p and hsa-miR-125b-5p for protection, with the last being reduced in the SF samples, and (ii) hsa-miR-21-5p and hsa-miR-30b-5p for destruction, again reduced in the SF samples. Notably, SF led to the upregulation of two abundant protective miRNAs, hsa-miR-193b-3p and hsa-miR-92a-3p, while IFNγ reduced protective hsa-miR-221-3p. Concerning synovia, whose correlation with miRNAs is still in its infancy, a balance towards protection was identified with an overall genetic weight of 1.53% vs. 0.68%, with SF reducing protective hsa-miR-26a-5p. Eventually, for macrophages, four miRNAs were identified for both M1 and M2 phenotypes, although hsa-miR-24-3p and hsa-miR-222-3p tipped the balance towards anti-inflammatory features with an overall genetic weight of 21.73% vs. 2.85%. Therefore, for all three categories under study, the OA protective signals largely overcame the promoting inputs, with SF having the highest number of modulated miRNAs.

### 3.4. Identification of EV-miRNA Reference Genes (RGs)

Four stability algorithms (Genorm, Normfinder, BestKeeper, and the comparative Delta Ct method) were used to determine the most stable EV-miRNAs among the candidates in the first quartile of expression that did not show modulation (even if not significant) in any condition, or with a C_RT_ SD > 1 in at least one of the conditions (Table 4 for the best six performers and Supplementary Table S5 for the complete ranking list). Out of 21 selected EV-miRNAs, the best performers were: Delta Ct, hsa-miR-130a-3p (0.49), hsa-miR-19b-3p (0.49) and hsa-miR-25-3p (0.53); BestKeeper, hsa-miR-130a-3p (0.18), hsa-miR-19b-3p (0.21) and hsa-miR-199a-3p (0.23); Normfinder, hsa-miR-130a-3p (0.15), hsa-miR-19b-3p (0.16) and hsa-miR-25-3p (0.27); Genorm, hsa-miR-17-5p (0.13), hsa-miR-106a-5p (0.13) and hsa-miR-19b-3p (0.27). hsa-miR-29a-3p, hsa-miR-222-3p, and hsa-let-7b-5p lay in the last positions of the ranking for all algorithms, with BestKeeper having hsa-miR-574-3p and hsa-miR-106a-5p at the bottom. Eventually, the geometric mean (Geomean) of each putative RG weight across the four algorithms was calculated, and the most stable RG was considered to be the RG with the lowest value. hsa-miR-130a-3p ranked best (1.5), while hsa-let-7b-5p was in last position (20.5).

## 4. Discussion

In this work, EVs and embedded miRNAs from adipose-derived MSCs, cultured under OA-mimicking conditions and characterized with the same technical workflow and platform, were compared. Different culturing conditions affected EVs size and release, while miRNA cargo shared conserved cartilage and synovia-protective and pro-M2 macrophage-polarizing features, with OA synovial fluid treatment able to overall modulate miRNA fingerprint although only a reduced fluctuations of OA-related miRNAs emerged.

In MSC field, EVs gained attention for mainly two reasons. With EVs being among the leading effectors of MSCs regenerative capabilities [6], on one hand they are studied to understand how MSCs may work for the different clinical needs. On the other hand, and as a consequence, EVs are envisioned as cell-free alternative [34] to be used directly or engineered to boost their potential. For both of these investigations, several common questions have arisen, such as the assessment of: (i) EVs’ release and properties; (ii) cargo composition tailored to clinical needs; (iii) cargo modulation both in vivo and under in vitro conditioning; and (iv) reliable reference molecules to allow the quantification of single therapeutic factors. Regarding the first point, although no differences appeared for EV size, inflammatory priming (high levels of INFγ and low levels of OA mimicking) led to an increase in EV secretion (Figure 1A). This is of particular importance for clinical EV production, since a major pitfall and restriction of the GMP setting is the need of the large expansion of cells, resulting in increased time, workforce and eventual expenses for large-scale EV purification [35]. A recent case study estimated the cost of GMP production to range between EUR 20,000 and almost EUR 200,000 when producing a cell batch [36]. This suggests a further increase in cost when EVs are produced due to additional purification and release tests. Therefore, increasing the number of doses per batch, or reducing the number of passages and therefore days of GMP activity would be of great benefit in view of clinical translation and the economic sustainability of this therapy. With respect to OA SF culturing resembling in vivo conditions, a consistent (*p*-value 0.0826) reduction in EV release was observed (Figure 1A). This implies that data obtained in vitro with a classical medium could overestimate ASC-EVs’ impact on target tissues in OA joints. In fact, it was recently demonstrated in a 3D physiological-like microenvironment that synoviocytes and chondrocytes incorporate only a few thousand MSC-EVs in a day [37] and that, in synoviocytes, a direct relationship between incorporation efficiency and EV to cell ratio is present [38]. Thus, future studies in vivo or in patient are needed to understand whether MSC potential through EVs observed in vitro, at least regarding particles release, can recapitulate clinical impact.

In relation to the first point, the deep characterization of EV cargo tailored to clinical requirements is mandatory. This analysis supports the previous reports [15,16,17], with the majority of OA-related miRNAs being constant in their amount between treatments and involved in synovia and cartilage protection, as well as anti-inflammatory M2 macrophage polarization. This emphasizes the need for sifting identified molecules through the sieve of the disease under study. Moreover, to avoid a general definition of target pathways and tissues, for OA-related bioinformatics analysis, we considered only miRNAs with a solid and experimentally validated description of their role in OA-associated tissues and cells, discarding all information related to in silico predictions. We are aware that this choice could reduce the overall prediction of analyzed miRNAs, but we are convinced that only an experimentally driven data analysis, especially if relying on bioinformatics tools, can sharply define miRNA’s role in a given condition. Our data have shown that, on a global level, both IFNγ and SF lead to the distinction of samples in both PCA and hierarchical clustering analyses (Figure 2A,B), suggesting a potential divergent impact on the target disease, i.e., OA for this study. Nevertheless, there were fine-tuned differences resulting from the modulation of the most abundant miRNAs, and a greatly reduced influence emerged for both OA-related miRNAs and their OA targets. Very often, a differential miRNA expression in the range of the two-fold maximum was present, with very few miRNAs increasing or decreasing by a factor higher than four. The most impactful miRNA was hsa-miR-125b-5p, which was reduced in SF samples, and which directly targets several OA-destructive molecules (TNFα, IGF2, ANGPT2, MMP2 and APC) and has an indirect protective role on cartilage. Despite suggesting a reduced protective impact for SF EVs, the upregulation of other miRNAs, such as hsa-miR-193b-3p targeting the ECM-degrading enzyme PLAU and regulating chondrocyte inflammation by repressing TNFα expression, rebalances the overall EV impact. These data highlight that, although low-grade fluctuations may influence the overall fingerprint, EVs released from ASCs are hardly affected by culturing conditions, as previously shown for bone marrow MSCs primed with hypoxia or IFNγ, which had only a limited effect on the EV-miRNA landscape [39]. This opens the question as whether in vitro priming is a preferable step to increase clinical-grade EV potency—rather than loading EVs with high amounts of disease-specific miRNAs [40,41]—and suggests that data collected under normal culturing conditions can nicely recapitulate the in vivo overall message, at least for OA. Moreover, together with miRNA role in the target disease, another way to understand miRNA impact on diseased tissues and cells will be possible when their mRNA fingerprint will be available together with more comprehensive miRNA-mRNA interaction definitions as shown for the impact of new drugs and medicines [42]. Additionally, a point of discussion is the effect of FBS starvation on EVs and their content after cell preconditioning, which may reduce high-level modulation. Furthermore, another intriguing question is whether the beneficial effects of preconditioning on MSCs’ biological activity [43], at both cell and whole secretome levels, rely on EV miRNAs or on other factors that are more heavily modulated, as we have shown for soluble mediators after IFNγ [15] or OA-SF [17] treatments. Thus, future studies are needed since, to date, rigorous data on the sharp separation of secretome molecules/particles without interspecies contamination are largely missing.

We are aware that a main limitation of this work is the reduced number of miRNAs under analysis. We prefer to study well-characterized molecules in order to attribute reliable biological significance. In May 2022, the number of described miRNAs increased to 38,589 (https://www.mirbase.org/, accessed on 1 May 2022) [44], and it is presumable that future studies will be needed to increase the knowledge on EV-shuttled molecules. In this context, a wider miRNA detection analysis coupled with an increased knowledge of their role in OA will be the basis for the future expansion of this study. Moreover, qRT-PCR was selected as the technology for miRNA detection due to its ease of use and scalability for single marker identification in the context of potency and release assays of clinical EV batches. For these reasons, hsa-miR-130a-3p—which was identified in this work as having the best RG—will be useful for both research and clinical approaches, as it is stable across several conditions and among the most abundant miRNAs of the EV cargo. This miRNA has been reported as directly and indirectly involved in pathways and molecules related to OA (Table 2 and Table 3). Nevertheless, we believe that a role of a RG in the pathology under analysis does not affect its suitability, presumably being just a matter of time to have reports about involvement of miRNAs without, to date, a known role in the given disease. Eventually, bioinformatics was conducted relying on miRNAs reported to have an experimentally validated role in OA tissues and cells and discarding in silico and not validated predictions. For these reasons, although we have greatly clarified the impact of ASC-secreted EVs and shuttled miRNAs on OA, readouts on preclinical and possibly clinical samples for confirmatory tests able to validate the bioinformatic-predicted outcomes are needed to define EV-miRNAs’ therapeutic potential. From this perspective, it is mandatory that results published in different studies rely on a conserved EV-miRNA fingerprint, regardless of laboratory workflow, age, or harvest site. Of note, high agreement (>70% match in detected EV-miRNAs) was observed between the results obtained in the present work, using ASCs from abdomen liposuction, with both a previous report of our research group with ASCs obtained from a local hip fat deposit [45] and a recently published manuscript where ASCs were similarly isolated from abdomen lipoaspirate [46]. Moreover, in these three studies, age, gender, and isolation techniques were not homogeneous, suggesting that the ASC-EV landscape is largely conserved, regardless of donor characteristics and technical workflow, allowing for largely reproducible and reliable results in mechanism studies on target tissues and cells aimed at confirming in silico predictions. In this field, few studies are present to date. Our group previously showed that ASC-EVs (obtained from three donors including ASC1 and 2 of this study) reduced the expression of pro-inflammatory cytokines/chemokines and metalloproteases in a chronic model of synoviocyte inflammation [38], including CCL5, IL6, and MMP1, thus confirming the bioinformatics-based modulation prediction in the herein presented study (Table 2). Zhao et al. have confirmed IL6 downregulation in synoviocytes, together with TNFα [47]. Regarding macrophages, Zhao and colleagues [48] showed that ASC-EVs were able to induce M2 macrophage polarization and mitigate inflammatory responses, as evidenced by a marked decrease in the levels of several factors including TNFα, again confirming our prediction. Moreover, M2 macrophages induced by ASC-EV promoted ASC proliferation, suggesting—in view of OA treatment—a general effect on stromal/progenitor populations similar to those present in synovium [49] and cartilage [50]. A similar result on macrophage biological and cellular processes was observed by Zhu et al., where M1 cells upon treatment with ASC-EVs became M2-like with a reduction in IL6 secretion, as herein predicted. Additionally, ASC-EVs were reported to alter both macrophage metabolism by inhibiting NF-κB abundance and signaling [51] (target of ASC-EV-embedded hsa-miR-138-5p)—initiating the inflammatory cascade and driving the production of IL1β, IL6, TNFα (all predicted in Table 2)—and other pro inflammatory cytokines [52], and as a consequence, cell–cell communication towards target cells. Eventually, ASC-EVs also showed protective effects on chondrocytes, confirming our prediction on the molecular level. Tofiño-Vian and colleagues reported in OA chondrocytes and cartilage explants that ASC-EVs reduced the production of inflammatory mediators, including TNFα and IL6 [53]. Again, NF-κB was an ASC-EV target, giving more ground for its involvement in modulating cellular and metabolic processes in target cells. Consistently, in inflamed chondrocytes, ASC-EVs were shown to downregulate COX2, mPGES1, iNOS and NO production, thus preventing the downstream induction and activation of MMPs and the inhibition of ECM synthesis by OA [53]. As a consequence, ASC-EVs promoted the expression of collagen II and reduced the abundance of MMP13, another NF-κB-regulated molecule. The reduction in MMP13, as well as MMP1/3, and the increase in COL2A1 were confirmed in an independent work on OA chondrocytes treated with ASC-EVs [54]. Altogether, these in vitro results suggest that ASC-EVs may modulate target cells both at the single mRNA/protein level with embedded miRNAs and on a more general level through an influence on biologic and metabolic processes, and such a paradigm has been confirmed in in vivo models. Fazaeli and colleagues reported that ASC-EVs ameliorated disease in a ciprofloxacin-induced OA mouse model, with reduced levels of collagen I and increased amounts of collagen II [55]. Similarly, the intra-articular injection of ASC-EVs attenuated OA progression and protected cartilage from degeneration in both a monosodium iodoacetate rat and a surgical destabilization of the medial meniscus mouse models, together with the inhibition of the infiltration of M1 macrophages into the synovium [54]. Therefore, ASC-EV preclinical data strongly support the in silico prediction for both single molecule and overall target cell homeostasis, although we are aware that a comprehensive in vitro model encompassing all OA-related cell types and tissues at the same time, as possible with next-generation experimental settings such as microfluidics, together with more data from in vivo reports, are needed. Such results will open the possibility of ASC-EV use in clinical trials for OA, such as the first one recently registered (https://clinicaltrials.gov/, accessed on 3 June 2022) using EVs from umbilical cord MSCs (NCT05060107).

## 5. Conclusions

Definitions of EV-shuttled signals and their modulation are crucial for MSC-related research, both for basic science and for use in clinical settings, either as therapeutics or as producers of cell-free products such as EVs or secretome. By sifting EV-miRNA datasets obtained from the same donors, this work showed overall cargo modulation under different OA-related stimuli, although with mild-to-moderate regulation in the most relevant OA-specific players, ending in fairly stable protective and healing signals and allowing for the faster clinical translation of these new cell-free therapies for joint diseases. The main difference appeared in the EV release rate in favor of inflammatory conditions, a crucial issue to compare in vitro and in vivo data or when clinical production is under consideration. Due to these premises, the future clinical perspective will be an increased because more aware use of both MSCs and their EVs for OA patients and, after disease-tailored analyses, for all those conditions where tissue homeostasis and inflammation management are required.

## Figures and Tables

**Table 1:** First-quartile differentially expressed EV-miRNAs with respect to C condition.

miRNA	Mean Fold vs. C			Sem Fold vs. C			Adjusted *p*-Value		
	I	OA	SF	I	OA	SF	I	OA	SF
hsa-miR-125b-5p	1.01	0.79	**0.36**	0.06	0.06	0.02	1.0000	0.2094	0.0043
hsa-miR-193b-3p	1.00	1.06	**3.34**	0.11	0.09	0.17	1.0000	1.0000	0.0152
hsa-miR-221-3p	**0.17**	0.76	0.69	0.04	0.04	0.11	0.0059	0.0955	0.3153
hsa-miR-99a-5p	0.67	0.85	**0.30**	0.06	0.05	0.05	0.0929	0.2712	0.0149
hsa-miR-100-5p	0.73	0.94	**0.35**	0.05	0.04	0.04	0.0941	0.8832	0.0138
hsa-miR-30c-5p	0.85	1.20	**0.24**	0.03	0.07	0.07	0.1483	0.2876	0.0226
hsa-miR-30b-5p	0.90	1.12	**0.34**	0.02	0.03	0.07	0.1597	0.1444	0.0362
hsa-miR-92a-3p	0.89	0.78	**3.55**	0.13	0.02	0.25	1.0000	0.0294	0.0278
hsa-miR-99b-5p	1.11	0.78	**0.18**	0.13	0.03	0.01	1.0000	0.0458	0.0006
hsa-miR-31-5p	1.65	1.12	**0.20**	0.32	0.11	0.01	0.5385	1.0000	0.0004
hsa-miR-214-3p	0.82	0.88	**3.41**	0.09	0.06	0.18	0.5675	0.5684	0.0174
hsa-miR-127-3p	1.09	0.93	**0.27**	0.10	0.03	0.03	1.0000	0.4370	0.0041
hsa-miR-26a-5p	1.70	0.90	**0.29**	0.36	0.05	0.03	0.5678	0.5240	0.0067
hsa-miR-27a-3p	**0.33**	0.87	0.50	0.02	0.08	0.02	0.0020	0.6694	0.0068
hsa-miR-152-3p	0.86	0.94	**0.48**	0.05	0.01	0.06	0.3411	0.0300	0.0352
hsa-miR-29c-3p	1.85	1.43	**0.36**	0.10	0.12	0.10	0.0397	0.1984	0.0661
hsa-miR-331-3p	1.68	1.00	**0.25**	0.28	0.04	0.04	0.4158	1.0000	0.0077
hsa-miR-27b-3p	**0.47**	0.86	0.80	0.00	0.08	0.13	0.0000	0.6132	0.8256
hsa-let-7c-5p	1.75	1.06	**0.27**	0.38	0.13	0.00	0.5592	1.0000	0.0000

Significant modulations are highlighted in bold.

**Table 2:** Soluble factors involved in the OA pathological state and the genetic weight of targeting the first-quartile EV-miRNAs. Cell type release for each factor is indicated with “X”.

Factor	Expressing Cell Type			First-Quartile EV-miRNAs			Factor Function
	Synoviocytes	Chondrocytes	Hla-Dr+	Total Genetic Weight %	Main Contributor	Modulation Main Contributor (*)	
***CYTO/*** ***CHEMOKINES***							
TNFα	X		X	12.7	hsa-miR-125b-5p	DOWN (SF) 2	Pro-inflammatory
IL1β	X		X	5.37	hsa-miR-21-5p		Pro-inflammatory
CXCL12	X		X	4.99	hsa-miR-221-3p	DOWN (I) 4	Articular cartilage matrix degeneration
IL1α	X		X	1.41	hsa-miR-191-5p		Inhibit proteoglycan synthesis by chondrocytes
IL6	X		X	1.18	hsa-miR-26a-5p	DOWN (SF) 2	Pro-inflammatory
CSF1	X	X		1.11	hsa-miR-130a-3p		Osteoclastogenesis enhancer, bone loss
CCL5	X		X	0.93	hsa-miR-214-3p	UP (SF) 2	Cartilage erosion
IL18	X		X	0.64	hsa-miR-130a-3p		Pro-inflammatory
TNFSF11	X	X		0.28	hsa-miR-106b-5p		Osteoclastogenesis enhancer, bone loss
***GROWTH FACTORS***							
TGFβ1	X	X	X	17.06	hsa-miR-24-3p		Cartilage homeostasis, high levels drive chondrocytes hypertrophy and synovial fibrosis
FGF1	X	X		15.54	hsa-miR-24-3p		Reduce cartilage matrix levels
IGF2	X	X		14.2	hsa-miR-125b-5p	DOWN (SF) 2	Promote cartilage matrix levels
ANGPT2	X		X	12.96	hsa-miR-125b-5p	DOWN (SF) 2	Abnormal angiogenesis in OA
VEGFA	X	X	X	12.29	hsa-miR-21-5p		Promote OA process
TGFβ2	X	X	X	6.75	hsa-miR-21-5p		Cartilage homeostasis, high levels released from joint tissue during OA development
CTGF	X	X	X	4.34	hsa-miR-30c-5p	DOWN (SF) 4	Promote osteophyte formation and ECM degradation
IGF1	X		X	3.09	hsa-miR-29a-3p		Promote chondrocyte anabolic activity
HGF	X		X	1.82	hsa-miR-199a-3p		Cartilage homeostasis, promote osteophyte formation and osteoblast abnormal mineralization
BDNF	X			1.21	hsa-miR-16-5p		Promote joint pain and inflammation
BMP2	X	X	X	0.95	hsa-miR-17-5p		Promote cartilage regeneration
FGF2	X	X	X	0.87	hsa-miR-152-3p	DOWN (SF) 2	Promote catabolic and anti-anabolic effects in OA joints
INHBB	X			0.68	hsa-miR-34a-5p		TGFB superfamily, upregulated in OA
BMP6	X			0.16	hsa-miR-22-3p		Promote chondrocyte proliferation
***PROTEASES & OTHERS***							
MMP2	X	X	X	18.29	hsa-miR-125b-5p	DOWN (SF) 2	Metalloproteinase involved in ECM degradation
MMP14	X	X	X	17.3	hsa-miR-24-3p		Metalloproteinase involved in ECM degradation
TIMP3	X	X	X	14.73	hsa-miR-21-5p		MMP inhibitor
APC	X	X		12.88	hsa-miR-125b-5p	DOWN (SF) 2	Activator of MMP
MMP1	X			6.43	hsa-miR-222-3p		Metalloproteinase involved in ECM degradation
PLAT	X	X		5.37	hsa-miR-21-5p		ECM-degrading enzyme
PLAU	X		X	4.70	hsa-miR-193b-3p	UP (SF) 2	ECM-degrading enzyme
ADAM17	X	X		2.44	hsa-miR-145-5p		Metalloproteinase involved in ECM degradation
TIMP2	X	X	X	1.53	hsa-miR-20a-5p		MMP inhibitor
ADAM8	X		X	1.34	hsa-miR-29a-3p		Metalloproteinase involved in ECM degradation
ADAMTS9	X			0.94	hsa-miR-29a-3p		Metalloproteinase involved in ECM degradation
ST14	X			0.24	hsa-miR-27b-3p	DOWN (I) 2	Serine proteinase involved in cartilage destruction
MMP9	X		X	0.18	hsa-let-7e-5p		Metalloproteinase involved in ECM degradation

* The number (2 or 4) indicates the ratio of modulation (>2 or >4, respectively). DOWN stands for downregulated with respect to C condition and UP for upregulated with respect to C condition.

**Table 3:** miRNAs involved in OA pathological state at cartilage, synovium, and macrophage levels.

miRNA	First-Quartile EV-miRNAs		miRNA Function
	Total Genetic Weight %	Modulation *	
***CARTILAGE***			
Protective			
hsa-miR-24-3p	15.54		Regulates chondrocyte senescence
hsa-miR-125b-5p	11.58	DOWN (SF) 2	Prevents aggrecan loss
hsa-miR-222-3p	5.05		Controls cartilage degradation via HDAC-mediated regulation of MMPs
hsa-miR-193b-3p	4.70	UP (SF) 2	Inhibits early chondrogenesis, regulates inflammation by repressing TNFα expression
hsa-miR-221-3p	3.83	DOWN (I) 4	Prevents ECM degradation
hsa-miR-92a-3p	1.89	UP (SF) 2	Anti-catabolic; increases collagen deposition
hsa-miR-145-5p	1.38		Regulates chondrocyte proliferation and fibrosis
hsa-miR-130a-3p	0.64		Anti-inflammatory, indirect suppressor of TNFα
hsa-miR-26a-5p	0.59	DOWN (SF) 2	Cartilage homeostasis promotes NF-κB p65 translocation
hsa-miR-320a-3p	0.48		Chondrocyte viability chondrogenesis
hsa-miR-17-5p	0.48		Induces autophagy
hsa-miR-199a-3p	0.38		Anti-catabolic
hsa-miR-27b-3p	0.24	DOWN (I) 2	Anti-catabolic; inhibits NF-κβ signaling
hsa-miR-210-3p	0.23		Inhibits NF-κβ pathway, anti-apoptotic, promotes chondrocyte proliferation and ECM deposition
hsa-miR-30a-5p	0.20		Cartilage homeostasis
hsa-miR-365a-3p	0.17		Prevents IL1β-mediated ECM loss
**TOT**	**47.38**		
**Destructive**			
hsa-miR-21-5p	5.37		Negatively regulates chondrogenesis
hsa-miR-30b-5p	2.03	DOWN (SF) 2	Autophagy inhibition, pro-apoptotic, ECM degradation
hsa-miR-145-5p	1.38		Cartilage degradation
hsa-miR-34a-5p	0.68		Apoptosis expression increases in chondrocytes exposed to H_2_O_2_
hsa-miR-16-5p	0.40		Cartilage degradation
hsa-miR-365a-3p	0.17		Mediates mechanical stress, pro-inflammatory
hsa-miR-138-5p	0.14		Promotes cartilage degradation
**TOT**	**10.17**		
***SYNOVIUM***			
**Protective**			
hsa-miR-29a-3p	0.94		Targets VEGF and suppresses ECM production
hsa-miR-26a-5p	0.59	DOWN (SF) 2	Targets COX2 to reduce Bcl2, IL6, TNFα, and IL8 expression
**TOT**	**1.53**		
**Destructive**			
hsa-miR-34a-5p	0.68		Promotes inflammatory mechanisms and oxidative stress
**TOT**	**0.68**		
***MACROPHAGE***			
**M2**			
hsa-miR-24-3p	15.54		Promotes M2; blocks M1
hsa-miR-222-3p	5.05		Promotes M2
hsa-miR-34a-5p	0.68		Promotes M2
hsa-let-7b-5p	0.46		Promotes M2
**TOT**	**21.73**		
**M1**			
hsa-miR-145-5p	1.38		Promotes M1
hsa-miR-130a-3p	0.64		Promotes M1; blocks M2
hsa-miR-26a-5p	0.59	DOWN (SF) 2	Blocks M2
hsa-miR-27b-3p	0.24	DOWN (I) 2	Promotes M1; blocks M2
**TOT**	**2.85**		

* The number (2 or 4) indicates the ratio of modulation (>2 or >4, respectively). DOWN stands for downregulated with respect to C condition and UP for upregulated with respect to C condition.

**Table 4:** Top 6 positions in the RG ranking of the stable first-quartile EV-miRNAs ordered following comprehensive ranking.

	Delta CT		Bestkeeper		Normfinder		Genorm		Comprehensive Ranking	
	SD		SD		SV		M		Geomean	
hsa-miR-130a-3p	0.49	(1)	0.18	(1)	0.15	(1)	0.33	(5)	1.5	(1)
hsa-miR-19b-3p	0.49	(2)	0.21	(2)	0.16	(2)	0.27	(3)	2.2	(2)
hsa-miR-25-3p	0.53	(3)	0.31	(5)	0.27	(3)	0.29	(4)	3.7	(3)
hsa-miR-199a-3p	0.54	(4)	0.23	(3)	0.30	(4)	0.37	(6)	4.1	(4)
hsa-miR-17-5p	0.58	(5)	0.40	(17)	0.40	(5)	0.13	(1)	4.5	(5)
hsa-miR-106a-5p	0.61	(8)	0.45	(21)	0.44	(7)	0.13	(1)	5.8	(6)

In brackets () the position in the ranking of each method.

## Data Availability

Data for the current analyses were retrieved from previously published datasets https://osf.io/2sg9e/ (accessed on 1 February 2022), https://osf.io/kjgxq/ (accessed on 1 February 2022) and the supplementary material of [17]. All raw data are now available at https://osf.io/usf84/?view_only=cc250bb06662429ca17332bc60ba339a (generated on 26 May 2022).

## Supplementary Materials

The following supporting information can be downloaded at: https://www.mdpi.com/article/10.3390/pharmaceutics14071400/s1. Figure S1: ASC morphology in control (CTRL) and synovial fluid (SF) conditions; Figure S2: Biological processes defined by first-quartile EV-miRNA targets, both total and depending on I or SF condition-modulated players; Table S1: Normalized EV-miRNA C_RT_ values across samples and conditions; Table S2: Amount of fold change of EV-miRNAs in I, OA and SF conditions with respect to C condition; Table S3: First-quartile EV-miRNA-validated targets; Table S4: First-quartile and I/SF condition-modulated EV-miRNA-validated targets. Table S5: Complete RG ranking of stable first-quartile EV-miRNAs ordered following comprehensive ranking.
