# Supplementary figures and images for: Joint Tissue Protective and Immune-Modulating miRNA Landscape of Mesenchymal Stromal Cell-Derived Extracellular Vesicles under Different Osteoarthritis-Mimicking Conditions
Source: Pharmaceutics. 2022 Jul 2;14(7):1400. doi: 10.3390/pharmaceutics14071400 (PMC9321932; doi:10.3390/pharmaceutics14071400)

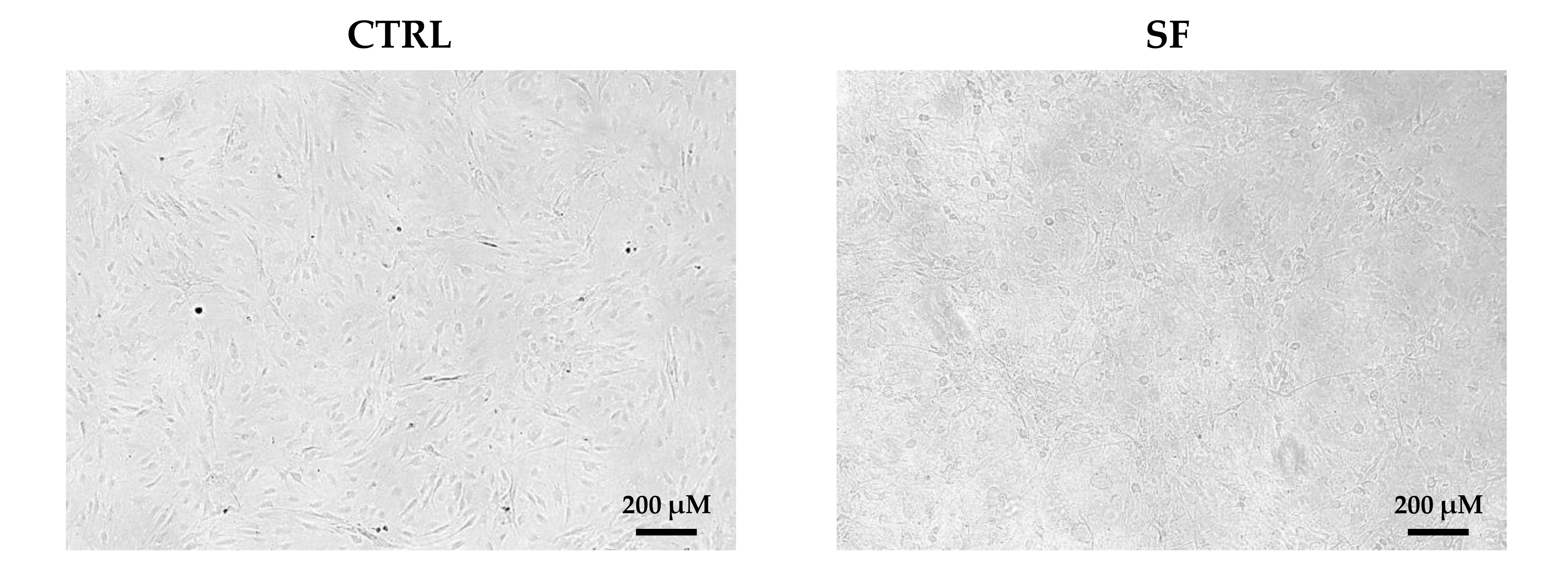

Supplement: Supplementary file 1 [file pharmaceutics-14-01400-s001.zip › Supplementary Figure S1_Rev - Pharmaceutics.tif]

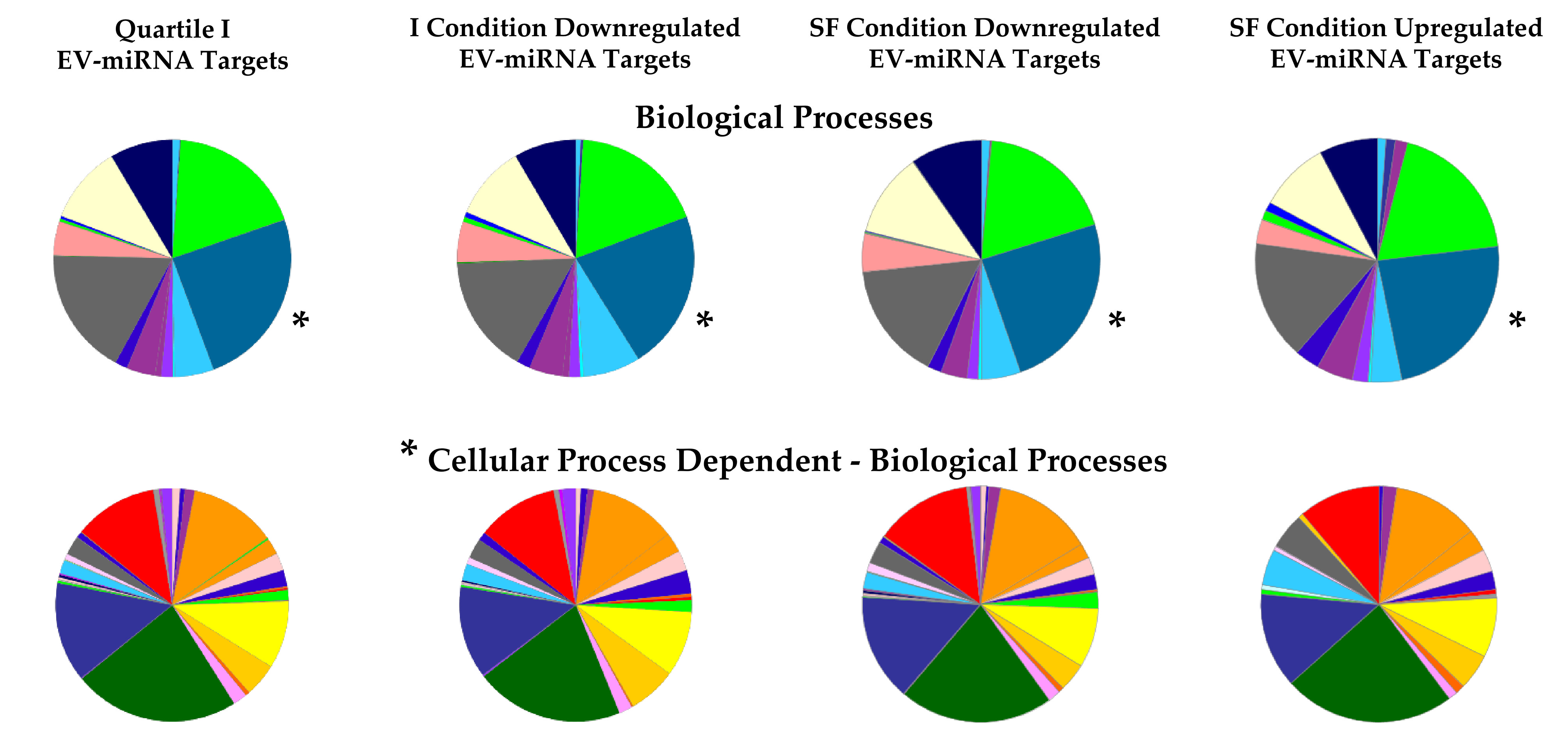

Supplement: Supplementary file 1 [file pharmaceutics-14-01400-s001.zip › Supplementary Figure S2 Rev3 - Pharmaceutics.tif]
